# Purity Analysis of Highly Purified Materials by Time-Temperature Cryometry

**DOI:** 10.6028/jres.067A.025

**Published:** 1963-06-01

**Authors:** Gaylon S. Ross, Herbert D. Dixon

## Abstract

Visual observation of the freezing and melting of compounds in cells used for the determination of purity has uncovered some heretofore unexpected behavior. This behavior has been correlated with certain difficulties experienced in the measurement of purity, particularly when the sample is very pure. Means for partially reducing these difficulties are proposed and procedures for increasing the accuracy of purity measurements are described.

## 1. Introduction

The usefulness of cryometry as a method of determining the purity of a compound has been well established. The time-temperature apparatus and technique developed by Rossini and coworkers [1]^1^ have been widely and successfully used. However, several years ago both Cines [2] and Mathieu [3] published comparisons between the results as obtained by the time-temperature techniques and those obtained by the use of a precision adiabatic calorimeter. Samples of various compounds were analyzed and serious differences between the two methods were found to exist. The differences in the results obtained by the two techniques were particularly significant when the more highly purified materials were analyzed. In these comparative studies, there appeared to be reasonable evidence supporting the view that, under the experimental conditions employed, the results obtained by using the adiabatic calorimeter were more reliable.

Enclosed freezing point cells [4], improved sample transfer [5], and a new method of analyzing data [6] have greatly improved the precision and accuracy of the time-temperature cryometric analysis.

This paper describes techniques used to explore further the utility of time-temperature cryometry into the region of highly purified materials, an area wherein previous work exhibited the most serious discrepancies. It will be seen from section 5, table 1, that the apparatus and techniques described give highly accurate results when used in the determination of purity of simple organic compounds.

## 2. The Freezing-Melting Curve

The apparatus and general experimental details discussed in this paper are modifications of those discussed in references [1, 4, and 5]. The success of any time-temperature cryometric determination of purity is dependent upon introduction of the sample without contamination, establishment of good thermodynamic and thermal equilibrium during the freezing process, and proper analysis of the experimental data. Under the experimental conditions imposed, the time-temperature freezing curve should be hyperbolic, and when materials of moderate purity are analyzed in the apparatus and by the procedures described in [4] the freezing curve exhibits normal behavior. However, with high purity material (99.99+percent), after initial recovery from supercooling, the observed temperature continues to rise until a large fraction (approximately 50 percent) of the sample is frozen. Beyond this point, the temperature falls rapidly. If the sample is melted after being about 35 percent frozen, the melting experiments produce the normal hyperbolic curves. These melting curves may be used to establish the sample purity, but in general such determinations are less accurate than those obtained from a well-performed freezing curve.

**Figure 1:**
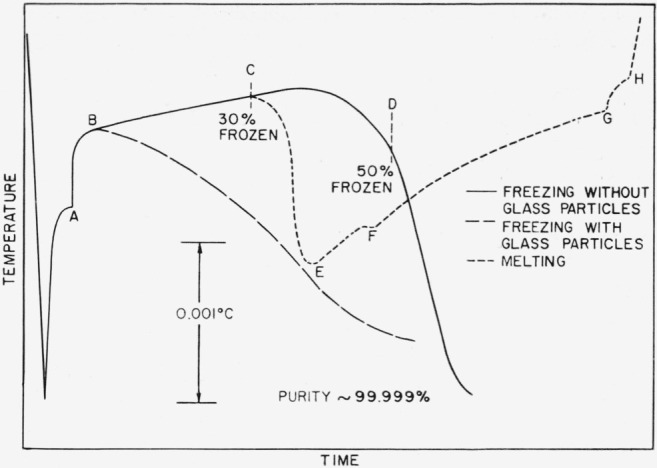
Typical freezing and melting curves for sample of high purity, with and without addition of glass particles. A, change in amplification. B, recovery from supercooling nearly complete. C, melting experiment started. D, bridging between thermometer well and outer cell walls, producing rapid fall of temperature. E, low point on melting curve. F, disruption of curve due to inclusion of solid from above liquid level. G, major portion of crystals melted. H, small crystalline mass in bottom of cell mixes with main body of liquid.

These anomalies in the freezing curves of highly purified materials have been observed for several years. At first they were thought to be associated with specific compounds, but as more materials of high purity were analyzed, it became strongly indicative that the unusual behavior was a result of the purity itself. Figure 1 depicts the general type of phenomenon observed during freezing and melting as described previously. This type of behavior has been observed with highly purified samples of benzene, titanium tetrachloride, carbon tetrachloride, dimethylphthalate, dichlorostyrene, isopropyl alcohol, certain metallo-organic compounds, and various isomers of dichloroethylbenzene.

Since the accuracy of the time-temperature cryometric measurement in the stirred cells is so dependent upon the ability to analyze the freezing curves, an investigation into the causes of the abnormal character of such curves was undertaken. For this work, unsilvered cells were used so as to permit visual observation of the freezing process. Samples of materials ranging in purity from 99.0 percent to 99.999 percent were used. It was observed that crystallization in the stirred cells always commenced on the inner surfaces of the sample cell. A thin sheath of crystal was formed on these walls, and probably on the thermometer well and the stirrer also. There were, however, important differences between the observed behavior of the moderately pure and the very pure samples. In the case of the less pure samples, the sheath was opaque and porous. The stirrer quickly dislodged it from the cell walls and suspended the crystals in the liquid. As crystallization proceeded, these crystals remained suspended in the liquid. The liquid quickly became opaque and gave the appearance of having a very large number of small crystals in suspension.

Contrariwise, with the very pure materials, the sheath was a clear hard mass which was not dislodged by the stirrer action. Instead, it continued to grow from the wall and, encroaching upon the stirrer space, the crystal was strong enough to stop the stirrer. The crystalline mantle surrounding the thermometer well also continued to grow, but the dendritic growth disappeared. Because of poor stirring the dendritic crystals sank to the bottom and were incorporated into the crystalline mass growing there. As crystallization continued, the crystal sheath adhered tenaciously to the thermometer well. A typical time-temperature freezing curve resulting from this type of freezing behavior is shown in figure 1. An approximate temperature scale is shown, corresponding to the temperature changes which occur when a material of high purity, 99.999 percent, is frozen and melted.

The melting process was also watched. If the sample was warmed as it reached point C (fig. 1), the first effect was the melting of the outer crystalline mantle. As this solid became detached from the cell walls and was included in the liquid, much better stirring was achieved. During this initial melting period the crystalline mantle around the thermometer also became detached. At point E, the bulk of the solid has become mixed with the liquid, and continued introduction of heat caused the solid-liquid mixture to melt under conditions which approach thermodynamic equilibrium. The inclusion of large crystal masses during this melting process caused serious discontinuities in the melting curve which made analysis difficult. At point F, crystals which had been deposited above the liquid level during early stages of freezing became incorporated in the main liquid. From this point, the curve assumed the usual hyperbolic shape of the melting curve. At point G the crystalline material suspended in the liquid had completely melted. There was a slight inflection at H due to a small compact mass of crystal which had previously remained on the curved bottom of the cell. After this mass melted, the curve assumed the usual warming slope of the liquid.

One possible explanation of the abnormal behavior of the freezing curve was suggested by the above observations. After initial recovery from supercooling, a crystalline mantle was formed around the thermometer and also around the cell wall. Because of poor stirring, the mantle surrounding the thermometer was not dislodged. The thermometer was a source of heat, and it is probable that a small amount of the solid surrounding the thermometer was melted. This thin layer of liquid would be quite pure, having been formed by the melting of the very pure crystalline phase. Thermodynamic equilibrium between these two phases would occur at a higher temperature than the equilibrium between the solid and the more impure bulk liquid. The temperature of this two phase region surrounding the thermometer would continually rise until the equilibrium temperature was reached. However, at point D, the advancing crystal front from the colder outer wall joined the crystal comprising the thermometer mantle, and the temperature fell rapidly.

Because of this, attempts were made to modify the freezing behavior pattern of the highly purified materials. It was found that when one or two cubic centimeters of small glass particles (approximately 1×10^12^ particles per cubic centimeter) were added to the highly purified material (40 ml), freezing behavior similar to that obtained with the less-pure systems was observed. These particles, primarily borosilicate glass, were obtained from the sludge produced by a carbide, glass-cutting tool, and the smaller particles were separated by gravity-sedimentation techniques. They were cleaned in the same fashion as the other glass components of the cell and were placed in the cell at the time of assembly of the transfer manifold. When treated in this fashion, there was no evidence of a change of sample purity in spite of the enormous surface area. Precise details of this operation will be given later. Many different substances have been analyzed using this technique. The particles, providing they were wetted by the sample liquid, appeared to be capable of satisfactorily modifying the freezing behavior of the purest samples, producing a normal hyperbolic freezing curve. The precise role of these particles is not known. They appear to act as crystal supports, suspended in the liquid. The crystalline mantles are quickly broken up and the large amount of suspended crystal causes thermal equilibrium to be obtained. It is probable that other materials could be satisfactorily used for this purpose; samples of diatomaceous earth added to samples of carbon tetrachloride or benzene exhibited no apparent effect.

## 3. Data Processing

The end result of the time-temperature freezing experiment is, of course, the determination of purity of the sample. The thermodynamic relationships, the theoretical proof that the experimental curve should be hyperbolic, and the usual methods of fitting the curve are well known and will not be discussed here. Basically, the analyst is interested in determining two numbers, the freezing temperature of the particular sample and the freezing point of the sample had there been no impurity present. Both of these numbers are obtained by fitting the data to the equation of a hyperbola. Extrapolation of this curve to the intersection of the liquidus cooling curve provides the freezing point of the sample, and further extrapolation to the asymptotic value provides the temperature at which the sample would have frozen had there been no impurity present.

When one is analyzing several samples of the same material but of different purity levels, the freezing points of the individual samples will vary as a function of the impurity present, but the extrapolated value for $T_{f_{o}}$, the freezing point of the pure material, should be invariant. This rule provides a valid test for any curve fitting technique. With this as a criterion, several hundred freezing point curves were analyzed, using a series of rather arbitrary curve-fitting rules. As a result it was found that only within the limits of 10 percent and 30 percent frozen, was the value of the experimentally determined $T_{f_{o}}$ invariant with respect to the purity of the particular material. The need for the lower limit follows directly from the argument that there must be a critical ratio of crystal area to liquid volume before temperatures corresponding satisfactorily to thermodynamic equilibrium can be registered by the thermometer. The upper limit of fraction frozen is the result of increased stirring energy and decreased stirring efficiency. These limits, imposed by particular apparatus and experiment design, seem to hold for all materials analyzed to date.

Other corrections which must be considered by the analyst include factors such as the significant change in heat capacity of the sample on freezing. Because of the difficult theoretical evaluation of the effect of these corrections on the freezing point equation, perhaps the best and most elegant method of fitting the data to an empirical curve is that of Saylor [6]. In this method, a series of positive transparencies of hyperbolae whose asymptotic intersection produces an angle of from 90 to 95° are first made. These 4×5 in. transparencies are placed in a photographic enlarger. The recorded freezing curves are placed under the enlarger so that by increasing or decreasing the magnification, the projected curve can be made to coincide with the experimental curve. Special holders for the transparencies and the recorded freezing curves are used. They allow the movement of each independently, without changing the coordinate interlock between the two. When the “best fit” is obtained, the experimental curve and the coinciding projection are photographed using a 35 mm camera mounted adjacent to the projector. The developed film serves as a permanent record, and the enlarged print may be used to calculate the purity.

By applying the fitting limit rules described previously, and by selecting the correct hyperbolic transparency, the fitting and subsequent calculation of purity is made very easy. Since, usually only 30 to 35 percent of the sample is frozen, the liquid cooling slope is used. The errors introduced by this substitution are negligible.

## 4. Experimental Detail

When compounds are less than 99.9 mole percent pure, inadvertent contamination during transfer is a relatively minor problem. As long as the samples do not react with air or water and if the containers are reasonably clean and dry, little relative contamination results from pouring or pipetting the sample from one container to another. However, with materials which have a total impurity of less than 10 parts per million, water in the atmosphere or adsorbed on the walls of the container, cell, and transferring manifold can easily increase the amount of impurity by a factor of 10 or more. It is obvious that if complete exclusion of water and air is desired, the sample must be transferred by and analyzed in a closed, evacuated system. The following describes a technique for transferring high-purity samples from a glass ampoule, equipped with a breakoff tip, to the freezing-point cell.

### 4.1. The Transfer of Highly Purified Materials

**Figure 2:**
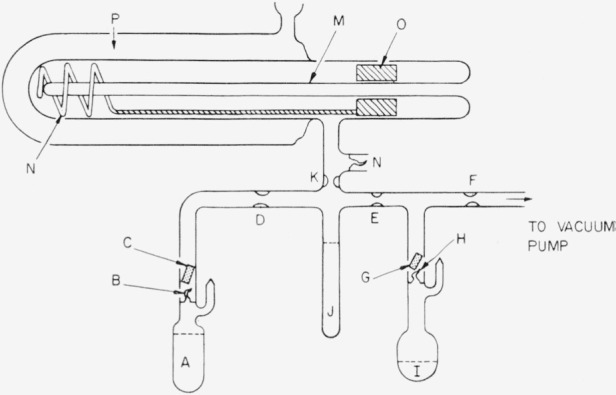
Freezing point cell and transfer manifold. A, degassed sample to be analyzed. Approximately 40 ml. B, H, breakoff glass seals separating contents of ampoule from rest of system. C, G, glass-enclosed iron bars. They are lifted by externally operated magnets and then dropped to rupture the seals. D, E, F, X, constricted portions of the glass manifold. L, side arm containing breakoff tip. Used to remove sample. J, graduated sample measuring tube. I, ampoule containing the degassed sample used as a preconditioning wash liquid. M, thermometer reentrant well. N, double helical platinum-iridium stirrer. O, platinum-enclosed, iron toroid connected to coiled stirrer by platinum-iridium rod. P, silvered vacuum jacket.

The transfers were carried out in all-glass evacuated systems containing no lubricated joints. Such a system is shown in figure 2. All parts were treated with hot chromic acid, water-rinsed, cleaned with warm, concentrated nitric acid, and finally rinsed several times with distilled water. The powdered glass, mentioned previously, was treated in the same fashion as the other glassware and placed in the cell prior to final assembly. The apparatus was then assembled and dried by evacuation to 10^−6^ mm Hg while being continuously heated at 150 °C for 24 hours. Previous experience [5] indicated that even after this treatment for the 24-hour period some water was still adsorbed on the inner glass surfaces.

The manifold was flame-sealed at constriction F after evacuation. Then the breakoff tip of ampoule I was broken and a portion of material similar in composition to the sample was distilled throughout the system. This wash liquid was returned to the ampoule after several hours of refluxing. As much of this material as possible was poured back into the ampoule by turning the entire apparatus manually. The remaining material was distilled into the ampoule by cooling container I with liquid nitrogen. This ampoule was then removed by sealing at E. The breakoff tip of ampoule A was broken, and the desired amount, usually 40 ml, was poured into the graduated tube J. After both A and J were cooled to liquid nitrogen temperature, the sample ampoule A was removed by sealing at D. The melted sample was poured into the cell and again frozen. Next the measuring tube J was removed by sealing at K. The breakoff tip L was used in removal of the sample from the cell after the analysis had been completed.

Previous experience [5] had shown that the pouring operation is to be preferred to distillation since there can be no change in composition induced in transfer by pouring, but if the impurity is sufficiently nonvolatile serious compositional changes could occur during a transfer by vacuum distillation. The powdered glass may selectively adsorb some of the impurity present in the wash sample. Whether this happens is not known but since the wash sample was similar in composition to the sample being analyzed there was no evidence of a detrimental adsorption-desorption process taking place when the sample is introduced.

### 4.2. The Freezing-Point Cell and Thermometric System

Freezing-point cells of the type described previously [4, 5] were used. The cells were closed so that samples could be analyzed in the absence of air and water vapor. The double helically wound stirrers were constructed of platinum-iridium, and stirring was achieved by means of a reciprocally moving external electromagnet which was coupled with the platinum-encased iron armature attached to the stirrer shaft. Figure 2 depicts a cell attached to the filling manifold. Constant-temperature freezing or warming baths were used. These baths were selected so that their temperatures differ from the freezing point of the sample by approximately 50°. The freezing rates were controlled by evacuation of the outer jacket of the cell to the appropriate pressure as determined by a Pirani gage.

A 25-ohm, bifilar, helical, glass-sheathed platinum resistance thermometer was used. The G–2 Mueller Wheatstone bridge used in this work was modified as described in [7]. The extreme stability which was achieved as a result of these modifications allowed automatic recording of the bridge output without the necessity of frequent zeroing. With this apparatus temperature differences of 0.00002 °C were detectable.

The freezing curves were recorded automatically in order to obtain a continuous record, to eliminate the tedious manipulation of dials, and to reduce operator bias. A considerable gain in both precision and accuracy was achieved by this recording method. The output from the nearly balanced Wheatstone bridge was amplified using a high-gain, d-c amplifier equipped with two duplicate sets of gain controls with external-switching mechanisms.

In our experience it has been extremely helpful to program automatically the freezing sequence. For this purpose the recorder was equipped with four switches. There were two limit switches at the opposite ends of the chart, a switch that was direction- activated by the appropriate direction change of the recorder pen, and a switch that could be preset to operate at any selected portion of the recorder span. These switches, together with a stopping switch and a repeating, adjustable cycle switch, were used to program the freezing-point experiment. Programing of the freezing or melting sequence can be a valuable, timesaving aid for the analyst who is engaged in the determination of purity of many samples of one material wherein the expected range of purity is quite small. The precision obtained by programing is always improved in comparison to the non-programed experiment sets. However, the basic accuracy is not necessarily improved, and the decision of whether to program or not should be made upon the number of replicate analyses expected.

**Figure 3:**
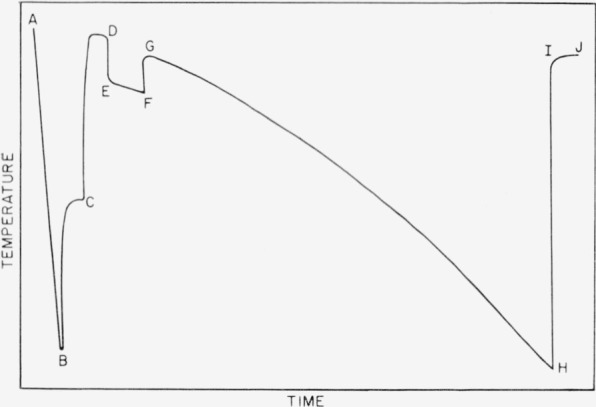
Programed freezing curve with benzene as the sample. A, Start of recorded experiment. Sensitivity—1 span/5 °C B, Spontaneous nucleation of sample. Direction switch activated. C, Recovery from supercooling nearly complete. Gain changed. Sensitivity—0.01 °C/span. D, Sensitivity check initiated. E–F, Change in bridge dial. G, Return of bridge dial to original setting. I, Current to bridge interrupted. J–H, Bridge zero.

The programing mechanisms may be of any variety of forms. Figure 3 depicts a real freezing curve showing the programing points. Through ABC one set of amplifier gain controls is adjusted so that a single recorder span corresponds to 5 °C. At point B the direction-sensitive switch is actuated. This starts a timed sequence of events. After a preset period of time (point C) the amplifier gain controls are switched to high sensitivity by advancement of the stepping switch. Again after a preset interval, the appropriate dial on the Mueller bridge is turned (point D) by one of a pair of electromagnets so as to determine the sensitivity. The selection of this point is important in that it should be prior to 10 percent frozen but after the highest recorded temperature has been reached. At time F the dials are automatically returned to their original position, by the other electromagnet. Point H represents approximately 35 percent frozen, and at this point the limit switch interrupts the bridge current, producing the zero plot from points I to J. The zero should not be taken in this fashion until the experiment is complete, since interruption of bridge current causes hysteresis effects which are not completely eliminated until approximately 10 minutes have elapsed.

## 5. Experimental Results

The most rigid test of the reliability of the general method described in this paper was imposed by participation in a cooperative project on purity control organized by the Commission on Physicochemical Data and Standards, International Union of Pure and Applied Chemistry. In this program 20 laboratories analyzed replicate samples of benzene to which controlled amounts of contaminant had been added. The list of participants included government, university, and industrial laboratories from 6 countries. The results of this investigation, as yet not published, were reported before the Calorimetry Conference in August 1961 at Ottawa, Canada. Table 1 lists the most probable purity value for each sample as decided by the Committee, together with the results as obtained by this method. It is sufficient to point out that there is excellent agreement from purity ranges of 99.999 mole percent down to 99.0 mole percent, and to note that among the several participants using the Glasgow, Streiff, Rossini technique, the methods suggested herein were the only ones which gave excellent agreement for all samples and, incidentally, agreed with those results obtained from application of precision adiabatic calorimetric techniques.

## 6. Summary

This paper discusses in detail the changes in technique which were necessary to extend the time-temperature cryometric method of purity determination to the realm of very highly purified materials. These changes in technique are summarized as follows:

1. The samples were transferred and analyzed in closed, evacuated systems.
2. The systems were rigorously cleaned, dried, and preconditioned with a portion of the sample to be analyzed.
3. Ground glass was added to insure the attainment of equilibrium in the freezing cell.
4. The time-temperature curves were automatically recorded.
5. When large numbers of replicate analyses were performed, the freezing experiments were programed.
6. Selection rules were devised to insure that the best portion of the time-temperature curve was used in the curve-fitting.
7. The optical projection method of curve-fitting was employed to eliminate operator bias and give more reliable results.

## Figures and Tables

**Table 1:** a

Sample designation	Most probable purity	Purity found	Estimated standard deviation (6 experiments per sample)
			
	*Mole %*	*Mole %*	
A–7	99.999	99. 999	±0.0002
A–16	99.999	99.999	0.0002
A–28	99.999	9.9999	0.0003
B–7	99.907	99.895	0.0018
B–16	99.907	99.894	0.0008
B–28	99.907	99.915	0.0012
C–7	99.797	99.808	0.005
C–16	99.797	99.796	0.001
C–28	99.797	99.760	0.004
D–7	98.822	98.824	0.011
D–16	98.832	98.932	0.025

a The complete results of this investigation will be published formally by the International Union of Pure and Applied Chemistry.

## Footnotes

1 Figures in brackets indicate the literature references at the end of this paper.

## References

[1] Glasgow AR, Streiff AJ, Rossini FD (1945). J Research NBS.

[2] Cines MR (1950). Physical Chemistry of Hydrocarbons.

[3] Mathieu MP (1953). Acad Roy Belg, Classe Sci Mem.

[4] Glasgow AR, Tenenbaum M (1956). Anal Chem.

[5] Glasgow AR, Ross GS, Horton AT, Enagonio D, Dixon HD, Saylor CP, Furukawa GT, Reilly ML, Henning JM (1957). Anal Chim Acta.

[6] Saylor CP (1957). Anal Chim Acta.

[7] Ross GS, Dixon HD (1960). J Research NBS.

